# Low factor XIII levels after intravenous thrombolysis predict short-term mortality in ischemic stroke patients

**DOI:** 10.1038/s41598-018-26025-z

**Published:** 2018-05-16

**Authors:** Edina Gabriella Székely, Katalin Réka Czuriga-Kovács, Zsuzsanna Bereczky, Éva Katona, Zoltán András Mezei, Attila Nagy, Noémi Klára Tóth, Ervin Berényi, László Muszbek, László Csiba, Zsuzsa Bagoly

**Affiliations:** 1 Division of Clinical Laboratory Sciences, Department of Laboratory Medicine, Faculty of Medicine, University of Debrecen, Debrecen, Hungary; 2 Department of Neurology, Faculty of Medicine, University of Debrecen, Debrecen, Hungary; 3 Department of Preventive Medicine, Faculty of Public Health, University of Debrecen, Debrecen, Hungary; 4 Department of Radiology, Faculty of Medicine, University of Debrecen, Debrecen, Hungary; 5 MTA-DE Cerebrovascular and Neurodegenerative Research Group, Debrecen, Hungary

## Abstract

In this observational study we investigated whether levels of factor XIII (FXIII) and its major polymorphisms affect the outcome of thrombolysis by recombinant tissue plasminogen activator (rtPA) in acute ischemic stroke (AIS) patients. Study cohort included 132 consecutive AIS patients undergoing i.v. thrombolysis within 4.5 h of symptom onset. Blood samples taken on admission, immediately after and 24 h after therapy were analyzed for FXIII activity and antigen levels. FXIII-A p.Val34Leu, p.Tyr204Phe, FXIII-B p.His95Arg and intron K(IVS11 + 144) polymorphisms were genotyped. Neurological deficit was assessed using the National Institutes of Health Stroke Scale. Intracranial hemorrhage was classified according to ECASSII criteria. Long-term functional outcome was defined at 3 months post-event by the modified Rankin scale. FXIII levels showed a gradual decrease immediately after thrombolysis and 24 h later, which was not related to therapy-associated bleeding. In a multiple logistic regression model, a FXIII level in the lowest quartile 24 h post-lysis proved to be an independent predictor of mortality by 14 days post-event (OR:4.95, 95% CI:1.31–18.68, p < 0.05). No association was found between the investigated FXIII polymorphisms and therapeutic outcomes. In conclusion, our findings indicate that FXIII levels 24 h after thrombolysis might help to identify patients at increased risk for short-term mortality.

## Introduction

Acute ischemic stroke (AIS) is a leading cause of death or disability in all developed countries^1^. Today, the most effective pharmacological therapy to improve functional outcomes is the lysis of thrombi using recombinant tissue plasminogen activator (rtPA) within 4.5 hours after the onset of symptoms^2^. Although this intervention has been used for many decades, little is known why in some patients thrombolysis is less efficient and which are the factors promoting non-desirable side effects in others. In a subset of patients thrombolytic therapy is inefficient due to the failure of recanalization of the closed vessel and no clinical improvement is observed^3^. On the other hand, approximately 7–8% of patients develop potentially fatal intracranial hemorrhage, despite the fact that those eligible for therapy are carefully selected to minimize bleeding risk. In most cases, these complications cannot be foreseen at the initiation of therapy and their occurrence remains unexplained. It is plausible that effectiveness as well as adverse effects of the thrombolytic agent could depend on hemostatic and fibrinolytic factors influencing the structure of thrombi and the susceptibility to lysis. With the introduction of mechanical thrombectomy in the management of acute stroke care, today it is becoming important to identify patients who are at risk of worse thrombolysis outcomes and would benefit from alternative treatments^4^.

Blood coagulation factor XIII (FXIII) is a key player in the last step of the coagulation cascade determining clot stability^5–7^. FXIII is a tetramer consisting of two, potentially active A subunits (FXIII-A) and two carrier/inhibitory B subunits (FXIII-B). Its activation occurs in the last step of the clotting cascade by thrombin and Ca^2+^ ^8^. The active form (FXIIIa) plays a crucial role in protecting the clot against prompt fibrinolytic degradation, which is achieved by at least three major ways. First, FXIIIa cross-links α_2_-plasmin inhibitor (α_2_-PI) and perhaps other plasma components to fibrin, which effectively hinders its proteolytic degradation by plasmin. Second, FXIIIa cross-links fibrin α-chains into a high molecular weight α-polymers, which most likely has a direct effect on the susceptibility of fibrin clot to lysis. Third, cross-linking of fibrin by FXIIIa decreases the binding of plasminogen to fibrin, and consequently, decreases plasminogen activation by tPA^9^. On the other hand, deficiency of FXIII leads to a life-long bleeding disorder and in many cases severe or fatal intracranial hemorrhage is a prominent feature of the disease^10^. FXIII has a number of common polymorphisms: five have been described in FXIII-A (p.Val34Leu, p.Tyr204Phe, p.Leu564Pro, p.Val650Ile, p.Glu651Gln), two in FXIII-B (p.His95Arg and c.1952 + 144 C > G in intron K)^6^. Today, growing evidence supports the role of FXIII as a risk factor in atherothrombotic disorders^9^. Among FXIII polymorphisms, the relation of FXIII-A p.Val34Leu with the risk of thrombotic diseases has been intensively investigated^5,6,9^. However, only few reports are available on the relation of ischemic stroke and FXIII levels and/or its polymorphisms, as most studies include patients with coronary artery disease and/or myocardial infarction^9,11^. Moreover, although the association of FXIII levels and/or its common polymorphisms with the outcome of thrombolytic therapy in AIS is biologically plausible, surprisingly, to date, only few studies involving relatively small cohort of patients have been published^12–16^. Here we aimed to determine the impact of FXIII levels measured before and during the course of thrombolysis as well as common FXIII-A and FXIII-B polymorphisms on therapeutic outcomes in an AIS population undergoing thrombolysis within the therapeutic time-frame.

## Results

### Study population

Baseline characteristics of patients and stroke outcomes are summarized in Table 1. A total of 132 consecutive AIS patients undergoing thrombolysis were included in the study. Mean age was 69.0 ± 12.2 years, and 58.3% were men. The median NIHSS score on admission was 8 (interquartile range: 5–14). According to the TOAST criteria, most patients suffered a large vessel thrombosis (n = 49, 37.1%). Average time from symptom onset to treatment with rtPA was less than 3 h in the cohort and the duration of thrombolysis was approximately one hour for each patient. In case of 7 patients intravenous thrombolytic therapy was supplemented with intraarterial thrombolysis according to the standard protocol; the final dose of rtPA and the duration of thrombolysis was not significantly different for these patients as compared to the rest of the study group.

**Table 1:** Baseline characteristics and outcomes of patients.

	Values
Number of patients	132
Male	77 (58.3)
Age (years)	69.0 ± 12.2
Risk factors	
Hypertension	101 (76.5)
Atrial fibrillation/flutter	35 (26.5)
Hyperlipidaemia	82 (62.1)
Diabetes mellitus	40 (30.3)
Previous stroke	42 (31.8)
Active smoker	32 (24.2)
Stroke severity	
NIHSS 0–5	37 (28.0)
NIHSS 6–10	48 (36.4)
NIHSS 11–15	21 (15.9)
NIHSS >15	23 (17.4)
Stroke etiology (TOAST)	
Small vessel occlusion	14 (10.6)
Large vessel thrombosis	49 (37.1)
Cardioembolic	27 (20.5)
Undetermined	42 (31.8)
Time from symptom onset to treatment with rtPA (min)	160 ± 46
Dose of rtPA (mg)	
Intravenous rtPA (n = 125)	68.2 ± 14.9
Intravenous and intraarterial rtPA (n = 7)	61.6 ± 14.7
Duration of thrombolysis (min)	64 ± 10
Imaging data (median; total range)	
ASPECT score on admission	10 (7–10)
ASPECT score at 24 h	9 (0–10)
Short-term functional outcome	
Favorable	53 (40.2)
No change	47 (36.6)
Unfavorable	21 (15.9)
Death	5 (3.8)
Undetermined	6 (4.5)
Long-term functional outcome	
mRS 0–1	46 (34.8)
mRS 2–5	34 (25.8)
mRS 6 (death)	29 (22.0)
Undetermined	23 (17.4)
Mortality by day 14	18 (13.6)
Intracerebral hemorrhage (ECASS II)	
aSICH	7 (5.3)
SICH	6 (4.5)

Data are expressed as mean ± standard deviation or number (percentage) unless otherwise stated. n, number; NIHSS, National Institute of Health Stroke Scale; TOAST, Trial of Org 10172 in Acute Stroke Treatment; rtPA, recombinant tissue plasminogen activator; ASPECT, Alberta Stroke Program Early CT score; mRS, modified Rankin score; ECASS II, European Cooperative Acute Stroke Study II; aSICH, asymptomatic intracranial hemorrhage; SICH, symptomatic intracranial hemorrhage.

Favorable short-term and long-term functional outcomes were observed in 53 (40.2%) and 46 (34.8%) cases, respectively. Stroke-associated mortality by day 7, day 14 and by the end of the 3^rd^ month post-event was observed in 5 (3.8%), 18 (13.6%), and 29 (22.0%) cases, respectively. Therapy-associated bleeding complication was detected in 13 cases (7 patients presented with aSICH, 6 patients with SICH). One patient has died following intracerebral hemorrhage.

### The influence of thrombolysis on FXIII levels

Mean FXIII activity and antigen levels before and during the course of thrombolysis are shown in Table 2. On admission (before thrombolysis) a considerable number of patients (n = 39, 29.5%; data not shown in Table) had FXIII levels above the upper limit of the reference interval (above 143% or 28 mg/l). FXIII levels showed a gradual decrease after thrombolysis; at 24 h post-lysis significantly lower FXIII levels were detected as compared to initial values. Strong correlation was observed between FXIII activity and FXIII antigen levels at all investigated occasions (time points A, B and C: Pearson r = 0.915, p < 0.001; r = 0.919, p < 0.001 and r = 0.917, p < 0.001, respectively; data not shown in Table).

**Table 2:** The influence of thrombolysis on factor XIII (FXIII) levels.

	Before thrombolysis (A)	Immediately after thrombolysis (B)	24 h after thrombolysis (C)	*P*-value* A vs. C
FXIII activity (%)	126.1 ± 36.1	124.7 ± 33.8	116.6 ± 35.0	0.034
FXIII-A_2_B_2_ (mg/l)	22.3 ± 7.6	21.4 ± 6.8	19.6 ± 6.4	0.002

Results are expressed as mean ± standard deviation. *ANOVA with Bonferroni post hoc test.

### Association of FXIII levels on admission with stroke characteristics

FXIII levels on admission showed no association with the severity of the stroke (Table 3) and no correlation was observed between FXIII levels and NIHSS scores on admission (Pearson r = −0.09, p = 0.28). FXIII activity was significantly higher in case of atherothrombotic stroke as compared to strokes of cardioembolic origin (Table 3). FXIII activity and antigen levels showed a weak negative association with the age of the patients (Pearson r = −0.299, p < 0.001 and r = −0.286, p < 0.001, respectively; data not shown in Table). FXIII antigen levels were significantly higher in active smokers vs. never-smokers (24.60 mg/l vs. 21.15 ± 0.9 mg/l, respectively, p < 0.05; data not shown in Table). No correlation was observed between FXIII levels and any measured routine clinical chemistry, hemostasis and hematology parameters. No correlation was observed between FXIII levels measured at any time points and data obtained from CT imaging analysis (ASPECTS; data not shown). No correlation was observed between FXIII activity/antigen levels measured on admission and the elapsed time between symptom onset to thrombolysis treatment (r = 0.081, p = 0.186 and r = 0.061, p = 0.251, respectively; data not shown in Table).

**Table 3:** Association between factor XIII (FXIII) levels before thrombolysis and the severity and etiology of the stroke.

*Severity of Stroke*					
	NIHSS 0–5 (n = 37)	NIHSS 6–10 (n = 48)	NIHSS 11–15 (n = 21)	NIHSS > 15 (n = 23)	*P*-value*
FXIII activity (%)	129.7 ± 39.9	126.2 ± 39.6	125.6 ± 26.4	120.5 ± 32.3	0.808
FXIII-A_2_B_2_ (mg/l)	23.1 ± 7.8	22.6 ± 9.5	21.8 ± 5.0	21.1 ± 5.4	0.805
***Etiology of Stroke***					
	**Small vessel infarcts (n = 14)**	**Large vessel thrombosis (n = 49)**	**Cardio-embolic (n = 27)**	***P***-**value**^†^ **small vessel infarcts vs. cardioembolic**	***P*** **-value** ^†^ **atherothrombotic vs. cardioembolic**
FXIII activity (%)	145.6 ± 33.4	130.5 ± 38.3	117.5 ± 24.0	0.038	0.012
FXIII-A_2_B_2_ (mg/l)	25.5 ± 7.4	22.8 ± 8.3	20.8 ± 4.8	0.160	0.058

Results are expressed as mean ± standard deviation. NIHSS, National Institute of Health Stroke Scale; n, number of patients. *Univariate analysis (ANOVA); ^†^ANOVA with Bonferroni post hoc test; atherothrombotic: small vessel infarcts + large vessel thrombosis.

### Association of FXIII levels during thrombolysis and therapy outcomes

FXIII levels before the initiation of the therapy (time point A) or immediately after thrombolysis (time point B) showed no association with short-term functional outcomes (Table 4). On the contrary, at 24 h after thrombolysis (time point C), significantly lower FXIII levels were detected in those patients who died within the first week following treatment. FXIII levels were remarkably low in these patients, approximately 50% as compared to values measured in patients with other outcomes. Mortality and low FXIII levels were not associated with bleeding complications. On the other hand, no difference was observed in the FXIII levels of patients with any other outcomes except for mortality (i.e. favorable outcome vs. no change or unfavorable outcome; data not shown). Patients experiencing bleeding after therapy were separately handled during the analysis due to different assumed underlying pathomechanisms.

**Table 4:** Association of factor XIII (FXIII) levels during thrombolysis with short-term functional outcomes.

	Favorable outcome (n = 51)	No change (n = 42)	Unfavorable outcome (n = 16)	Death* (n = 4)	Intracerebral hemorrhage (n = 13)	*P*-value^†^ death vs. all other outcomes	*P*-value^†,‡^ death vs. all other outcomes
Before thrombolysis (A)							
FXIII activity (%)	127.3 ± 35.2	128.2 ± 41.1	128.1 ± 26.3	100.0 ± 28.7	125.2 ± 36.9	0.129	0.340
FXIII-A_2_B_2_ (mg/l)	22.9 ± 8.9	22.4 ± 7.7	22.7 ± 4.4	18.3 ± 5.5	21.6 ± 5.99	0.165	0.416
Immediately after thrombolysis (B)							
FXIII activity (%)	123.1 ± 33.2	130.1 ± 35.9	123.3 ± 31.9	111.3 ± 18.8	119.3 ± 38.4	0.256	0.596
FXIII-A_2_B_2_ (mg/l)	21.2 ± 6.5	22.4 ± 7.8	20.3 ± 5.5	20.1 ± 4.2	20.8 ± 7.8	0.335	0.702
24 hours after thrombolysis (C)							
FXIII activity (%)	115.8 ± 28.2	122.0 ± 40.6	116.0 ± 33.8	60.0 ± 28.9	119.9 ± 40.4	0.003	0.009
FXIII-A_2_B_2_ (mg/l)	20.0 ± 5.3	19.9 ± 7.6	19.5 ± 5.8	9.7 ± 5.3	19.1 ± 6.9	0.006	0.014

Short-term functional outcomes were assessed at 7 days post-event. Results are expressed as mean ± standard deviation. n, number; favorable outcome: a decrease in NIHSS score by at least 4 points or to 0 by day 7; no change: a change in NIHSS score less than 4 points by day 7; unfavorable outcome: an increase in NIHSS score by at least 4 points by day 7; *stroke-associated death excluding death due to intracerebral hemorrhage; ^†^ANOVA with Bonferroni post hoc test; ^‡^adjusted to age and sex in the statistical model.

**Figure 1:**
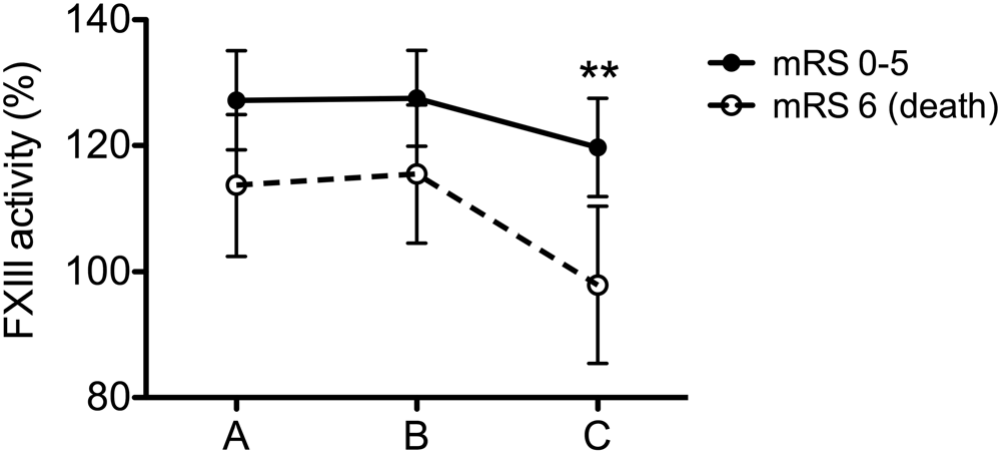
Changes in FXIII activity during thrombolysis in AIS patients of different outcomes by the end of the 3^rd^ month. FXIII activity levels are presented as mean (circles) and 95% confidence intervals (whiskers) before thrombolysis (**A**), immediately after (**B**) and 24 h after thrombolysis (**C**) in AIS patients of different outcomes by the end of the 3^rd^ month. Solid circles represent patients with mRS 0–5 (n = 80), open circles indicate patients who did not survive (mRS 6, n = 29). **p < 0.01 (Student’s t test).

Low FXIII levels were associated with mortality by day 14 post-event as well. When studying the baseline characteristics of patients grouped according to mortality by the end of the 2^nd^ week, it was found that besides the NIHSS on admission, only FXIII levels measured before and 24 h after thrombolysis were significantly different in the two groups (Table 5). Similar results were observed when FXIII levels were investigated in terms of functional outcomes at 3 months post-event (Fig. 1). FXIII levels 24 h after lysis were significantly lower in those patients who died by the end of the 3^rd^ month after the event (mRS 6). In order to test whether a low FXIII level measured 24 h after thrombolysis is an independent predictor of short- and long-term mortality of patients, backward multiple regression analysis for mortality was performed including all potentially relevant risk factors and confounders (Table 6). NIHSS score on admission was found to be an independent predictor of mortality by 14 days (OR: 1.12; 95% CI: 1.02–1.23, p = 0.013) and by the end of the 3rd month after the event (OR: 1.16; 95% CI: 1.05–1.28, p = 0.004). FXIII levels in the lowest quartile 24 h after thrombolysis were found to be an independent predictor of short-term mortality (OR: 4.95; 95% CI: 1.31–18.68, p = 0.018). On the other hand, low FXIII levels 24 h after thrombolysis did not prove to be an independent predictor of mortality at 3 months post-stroke (OR: 1.88; 95% CI: 0.55–6.41, p = 0.311).

**Table 5:** Demographic data and FXIII levels of patients grouped according to stroke-related mortality by 14 days post-event.

Variables	Survival (n = 114)	Death (n = 18)	*P-value*
Age, years	68.0 (58.0–79.0)	75.5 (63.0–83.3)	0.130
Male	67 (58.8)	11 (61.1)	0.851
Arterial hypertension	87 (76.3)	13 (73.7)	0.707
Atrial fibrillation	28 (24.6)	7 (38.9)	0.201
Hyperlipidaemia	73 (64.0)	9 (50.0)	0.254
Diabetes mellitus	32 (28.1)	8 (44.4)	0.160
Previous stroke	39 (34.2)	3 (16.7)	0.138
Active smoker	28 (24.6)	4 (22.2)	0.830
Serum glucose (mmol/L)	6.4 (5.5–7.7)	6.9 (5.6–8.4)	0.490
C-reactive protein (mg/L)	3.0 (1.5–5.6)	5.2 (2.2–9.6)	0.112
Antihypertensive therapy	75 (65.8)	12 (66.7)	0.942
Antiplatelet therapy	48 (42.1)	10 (55.5)	0.285
Time to treatment (min) with rtPA	157.0 (125.0–180.0)	144.0 (130.0–212.0)	0.605
Cardioembolic stroke	23 (20.2)	4 (22.2)	0.841
NIHSS on admission	8.0 (5.0–13.0)	14.0 (8.0–23.0)	0.005
ASPECT score on admission	10.0 (9.0–10.0)	9.5 (9.0–10.0)	0.591
FXIII activity (%) before thrombolysis	129.0 ± 36.0	107.4 ± 31.5	0.017
FXIII-A_2_B_2_ (mg/L) before thrombolysis	22.9 ± 7.8	18.6 ± 4.8	0.026
FXIII activity (%) immediately after thrombolysis	127.1 ± 33.7	110.9 ± 32.0	0.061
FXIII-A_2_B_2_ (mg/L) immediately after thrombolysis	21.8 ± 6.8	18.5 ± 6.4	0.056
FXIII activity (%) 24 h after thrombolysis	119.9 ± 33.6	91.3 ± 36.5	0.004
FXIII-A_2_B_2_ (mg/L) 24 h after thrombolysis	20.2 ± 6.2	15.1 ± 6.5	0.005

Values are given as mean ± SD or median (IQR) or number (percentage). FXIII, factor XIII; rtPA, recombinant tissue type plasminogen activator; NIHSS, National Institute of Health Stroke Scale; ASPECT, Alberta Stroke Program Early CT score.

**Table 6:** Predictors of short-and long-term mortality in the study group.

	OR (95% CI)	*P* value
Mortality by day 14 (n = 18)		
NIHSS score on admission	1.12 (1.02–1.23)	0.013
FXIII activity in the lower quartile 24 h after thrombolysis	4.95 (1.31–18.68)	0.018
Mortality by the end of the 3^rd^ month (n = 29)		
NIHSS score on admission	1.16 (1.05–1.28)	0.004
FXIII activity in the lower quartile 24 h after thrombolysis	1.88 (0.55–6.41)	0.311

Backward multiple regression model included age, sex, NIHSS score on admission, active smoking, C-reactive protein levels and the categorical variable of factor XIII (FXIII) activity in the lower quartile as measured from samples taken 24 h after thrombolysis (time point C). n, number of patients; CI: confidence interval; NIHSS, National Institute of Health Stroke Scale.

### FXIII levels are not associated with therapy-associated symptomatic or asymptomatic intracerebral hemorrhage

In patients suffering therapy-associated intracerebral bleeding complications (n = 13, Table 4), FXIII levels during thrombolysis were surprisingly similar to that observed in patients with other outcomes except for death. FXIII levels showed no significant difference at any measured time points between patients not experiencing hemorrhage and patients with SICH or aSICH (Supplementary Table S1). In this cohort, only one patient with therapy-associated hemorrhage had low FXIII levels (FXIII activity at time point A: 45.9%, B: 50.2%, C: 39.9%); heterozygous FXIII-A deficiency was ruled out in this patient by direct fluorescent sequencing of F13A1 gene (data not shown). Except for this single patient, FXIII levels were within or above the reference range at all measured time points in patients suffering symptomatic or asymptomatic bleeding as side-effect. One patient had died due to therapy-associated SICH by day 1, in this case, FXIII levels were also within the reference interval (FXIII activity at time point A: 107.7%, time point B: 104.5%).

### FXIII polymorphisms, stroke characteristics and thrombolysis outcomes

Allele frequencies of the common FXIII-A and FXIII-B polymorphisms were not significantly different in this AIS patient population as compared to a large cohort of population control group tested earlier^17,18^ (data not shown). In agreement with previous findings^6^, the FXIII-A p.Val34Leu, FXIII-A p.Tyr204Phe, FXIII-B p.His95Arg polymorphisms had no influence on FXIII levels (data not shown). Carriers of the FXIII-B intron K nt29756 G allele showed significantly lower FXIII levels as compared to non-carriers (FXIII activity: 114.5 ± 30.9% vs. 130.46 ± 36.9%, p = 0.021, respectively and FXIII antigen levels: 19.26 ± 1.2 vs. 23.5 ± 0.75, p = 0.004, respectively), but after adjustment to confounders (age, CRP, smoking), differences were not statistically significant among the two groups (data not shown in Table). None of the investigated factor XIII polymorphisms were associated significantly with stroke severity, unfavorable short-term or long-term outcomes of therapy, therapy-associated symptomatic intracranial hemorrhage and mortality (Table 7). In case of FXIII-A p.Val34Leu polymorphism, a trend could be observed showing that carriers of the Leu allele might be protected against unfavorable short-term outcomes (OR: 0.33; 95% CI: 0.09–1.10), but the association was not statistically significant (p = 0.072).

**Table 7:** Factor XIII (FXIII) polymorphisms, stroke severity and the outcome of thrombolytic therapy.

	FXIII-A p.Val34Leu	FXIII-B p.His95Arg	FXIII-B intron K nt29756 C>G
	OR (95% CI), *p-*value		
Stroke severity (NIHSS > 5)*	0.70 (0.32–1.54), 0.379	2.10 (0.73–6.03), 0.167	1.34 (0.56–3.25), 0.514
ICH^**†**^	2.72 (0.79–9.35), 0.112	0.17 (0.02–1.49), 0.111	0.41 (0.05–3.55), 0.673
SICH^**†**^	1.25 (0.17–8.80), 0.823	1.00 (0.09–10.97), 0.996	0.18 (0.01–3.34), 0.185
Unfavorable short-term outcome (ΔNIHSS ≥ 4)^**†**^	0.33 (0.09–1.10), 0.072	0.76 (0.22–2.66), 0.665	1.14 (0.38–3.33), 0.811
Unfavorable long-term outcome (mRS > 1)^**†**^	1.33 (0.50–3.52), 0.568	2.65 (0.85–8.26), 0.093	1.26 (0.45–3.50), 0.659
Mortality by 14 days^**†**^	0.61 (0.17–2.10), 0.434	1.84 (0.57–5.92), 0.305	1.12 (0.28–4.36), 0.871
Mortality by the end of the 3^rd^ month^**†**^	0.45 (0.14–1.44), 0.178	1.02 (0.30–3.42), 0.975	0.91 (0.29–2.82), 0.864

FXIII-A, factor XIII A subunit; FXIII-B, factor XIII B subunit; OD, odds ratio; CI, confidence interval; NIHSS, National Institute of Health Stroke Scale; ICH, intracranial hemorrhage (asymptomatic and symptomatic intracranial hemorrhage); SICH, symptomatic intracranial hemorrhage; ΔNIHSS, difference in NIHSS score by day 7; mRS, modified Rankin score. Short-term and long-term outcomes were assessed at 7 days and by the end of the 3^rd^ month post-event, respectively. *Adjustment in the statistical model to age and gender, ^**†**^adjustment in the statistical model to age, gender and NIHSS score on admission.

## Discussion

Despite the fact that activated FXIII plays a prominent role in the protection against fibrinolysis by plasmin, this is the first comprehensive study investigating FXIII activity and antigen levels during thrombolysis in AIS patients and the association of FXIII levels and polymorphisms with clinical outcomes. Here we show that FXIII activity and antigen levels decrease gradually during thrombolysis. The mechanism involved in such reduction of FXIII levels is not entirely clear. In theory, this phenomenon might be attributed to consumption and/or degradation of FXIII. A likely explanation of the considerable diminution of FXIII levels post-stroke is its continuous incorporation into the growing thrombus, leading to consumption due to ongoing activity of the coagulation system. This hypothesis was suggested by an earlier pilot study, where FXIII subunit levels (but not FXIII activity) were measured in a group of AIS patients^14^. In that cohort, 41 patients received thrombolysis by rtPA or urokinase and their results did not differ from AIS patients not receiving thrombolytic therapy (n = 23). Moreover, in another study that investigated the dynamics of FXIII levels during the early phases of acute myocardial infarction in 350 patients, a significant, transient drop of FXIII levels was observed in the majority of patients during the first five days post-event^19^. Similarly to our findings, the probability of early death was increased in those having the highest drop in FXIII levels (below 59.5%). As these patients did not receive thrombolytic agents, this phenomenon was attributed solely to consumption of FXIII associated with the evolution of infarction.

An additional mechanism for the decrease in FXIII levels observed in our study might be the proteolytic degradation of FXIIIa in the thrombus by plasmin or other proteases. Recently, it has been shown that activated FXIII, but not the zymogen form of FXIII is cleaved and inactivated by plasmin *in vitro*^20^. Less is known about this effect of plasmin *in vivo*. FXIII activity and antigen measurements performed in our study reflect circulating zymogen FXIII levels, which are not expected to be cleaved by plasmin. In fact, in our study FXIII activity and antigen levels measured before and immediately after thrombolysis were not significantly different, suggesting that plasmin has negligible effect on zymogen FXIII *in vivo*. On the other hand, incorporated FXIIIa might become degraded and inactivated by plasmin (and/or other proteases, e.g. proteases released from polymorphonuclear cells^21,22^) in the thrombus, attributing to the significant decrease observed in FXIII levels at 24 h post-lysis. Given the fact that the ELISA method used for FXIII-A_2_B_2_ determination in our study detects the intact FXIII tetramer complex only and no degradation products, we could not test the latter hypothesis and this must be investigated experimentally in future studies.

Using a logistic regression model including all potentially relevant risk factors, we found that a low FXIII level 24 h post-event is an independent predictor of short-term mortality (by 14 days). This result suggests that the reduction in FXIII levels directly relates to the pathomechanism of fatal stroke. This effect was found to be independent of the severity of stroke as measured by the NIHSS. Remarkably, those patients who died within the first week after stroke had unusually low FXIII levels the day after thrombolysis; with FXIII levels reaching only approximately 50% as compared to patients with better outcomes. Low FXIII levels as measured 24 h post-lysis were found to be associated not only with short-term but with long-term mortality (by the end of the 3^rd^ month post-event) as well. However, in the logistic regression model, low FXIII levels 24 h post-lysis did not prove to be an independent predictor of long-term mortality. Post-stroke mortality at a long-term is influenced by a number of factors including age, co-morbidities, etc. which could explain the loss of significance in this case.

Although it is biologically plausible, low FXIII levels were not associated with therapy-associated intracranial bleeding in our study, indicating that hemorrhagic complications must be associated with an etiology other than that linked to FXIII. This finding is in compliance with the few previously published reports^12,13^. FXIII levels were not associated with stroke severity in our patient cohort. As it has been found earlier^11^, we also showed that FXIII activity was significantly higher in case of atherothrombotic stroke as compared to strokes of cardioembolic origin.

In this study no association was found between any of the investigated major FXIII polymorphisms with stroke severity, unfavorable outcomes of therapy, therapy-associated symptomatic intracranial hemorrhage and mortality. In case of the FXIII-A p.Val34Leu polymorphism, an interesting trend was observed showing a protective effect against unfavorable short term outcomes in carriers of the Leu allele, but results did not reach statistical significance. No gender-specific differences with regard to the outcome of therapy were observed in case of any of the investigated polymorphisms (data not shown). Among the polymorphisms investigated in the study, only FXIII-B intron K nt29756 G allele was associated with lower FXIII levels, but after adjustment to confounders differences between carriers and non-carriers were not statistically significant. In a most recent study investigating patients with coronary sclerosis (CS) and/or myocardial infarction (MI), carriers of the FXIII-B intron K nt29756 G allele had significantly lower FXIII levels, and the presence of the allele provided significant protection against CS and MI in patients with fibrinogen in the upper tertile, which prevailed only in the presence of FXIII-A Leu34 allele^18^. In our study, the presence of FXIII-B intron K nt29756 G allele did not seem to have any impact on therapeutic outcomes, although due to the limited number of patients, it was impossible to perform subgroup analysis to seek for any synergistic effect between FXIII-A Leu34 and the FXIII-B intron K nt29756 G allele.

The study has limitations. Firstly, the study was designed to look for changes in FXIII levels during thrombolysis and to compare it with outcomes and was not designed to be a case-control study. Theoretically, we could compare FXIII levels in AIS patients receiving and not receiving thrombolysis. However, while the group of patients receiving rtPA is a highly selected patient group with strict inclusion criteria (e.g. time-frame from symptom onset to treatment, age limit, absence of effective anticoagulation, etc.), the group not receiving rtPA is a group not meeting the inclusion criteria by definition. The important baseline differences of the two groups and the heterogeneity of the group not receiving thrombolysis suggest that it might not be adequate to draw conclusions from the comparison of these groups.

Second, the sample size in this study is limited but representative for a single-center study. In fact, when comparing our study with previously published literature on this subject, our study included the largest cohort of patients^13–16^. Although the number of patients with symptomatic hemorrhage was small in this cohort, the lack of association of hemorrhage with FXIII levels was evident. It has to be noted, that although the single-centered design of the study has limitations regarding patient numbers, it gave us the advantage of assured unified sample handling, allowing reliable and reproducible results.

In conclusion, a low FXIII level 24 h after thrombolysis in AIS patients is an independent predictor of mortality by 14 days post-event. Testing FXIII levels 24 h post-lysis might help to identify patients who are at high risk of mortality and need altered therapeutic approach. Further studies including large number of patients are required to find out whether early selection of such patients could help to improve outcomes by providing better treatment strategies.

## Methods

### Study population

The study was designed as an observational study. Patients were enrolled between March 2011 and January 2013 in a single Stroke Center (Department of Neurology, University of Debrecen, Hungary). Study population included 132 consecutive patients admitted with AIS within 4.5 h of their symptom onset. All patients underwent intravenous (i.v.) thrombolytic therapy using recombinant tissue plasminogen activator (rtPA, Alteplase, Boehringer Ingelheim, Germany) according to the European Stroke Organization guidelines^5^. Patients or their relatives had been informed about the study and gave written informed consent. Ethical approval was obtained by the Ethics Committee of University of Debrecen, Debrecen, Hungary. All investigations and methods were performed in accordance with the relevant guidelines and regulations.

### Blood sampling and laboratory measurements

Peripheral blood samples were drawn from patients into vacutainer tubes. Tubes with no anticoagulant were used for routine clinical chemistry tests, tubes anticoagulated with K_3_-EDTA for complete blood count, tubes containing 0.109 M sodium citrate or CTAD (buffered citrate, theophylline, adenosine and dypiridamole) for hemostasis tests (Becton Dickinson, Franklin Lakes, NJ). Blood samples were collected on three different occasions: upon hospital admission (before thrombolysis) (time point A), immediately after the administration of rtPA infusion (64 ± 10 min, time point B) and approximately 24 h after the administration of thrombolytic therapy (time point C). Routine blood count and chemistry tests were performed only from samples collected at time point A. Chemistry tests included the measurements of serum ions, glucose levels, basic kidney function tests, liver function tests and high sensivity C-reactive protein (CRP) measurements (Roche Diagnostics, Mannheim, Germany). Prothrombin time (PT) and activated partial thromboplastin time (APTT) were determined at time point A while thrombin time (TT) was measured on all three occasions by routine methods (Siemens AG, Erlangen, Germany). Plasma from CTAD tubes was separated by centrifugation (1500 g, 20 min) and samples were stored at −70 °C until further analysis.

Plasma levels of FXIII activity were determined by ammonia release assay^23^ using a commercially available reagent kit (REA-chrom FXIII kit, Reanal-ker, Budapest, Hungary, reference range: 69–143%, CV: 3.8%). FXIII-A_2_B_2_ antigen levels were determined by a sandwich enzyme-linked immunosorbent assay (ELISA), comprising of a biotinylated monoclonal capture-antibody against the B-subunit and a peroxidase-labelled monoclonal tag-antibody against the A-subunit (reference range: 14–28 mg/l, CV: 2.0%)^24^. DNA isolation was performed from buffy coat of CTAD blood samples by QIAamp DNA Blood Mini Kit (Qiagen, Hilden, Germany). FXIII-A p.Val34Leu (c.103 G > T; rs5985), FXIII-A p.Tyr204Phe (c.614 A > T; rs3024477), FXIII-B p.His95Arg (c.344 G > A; rs6003) and FXIII-B Intron K (IVS11 c.1952 + 144 C > G; rs12134960) polymorphisms were determined by in-house developed real-time PCR methods^25,26^ using fluorescence resonance energy transfer (FRET) detection and melting curve analysis on a LightCycler® 480 instrument (Roche Diagnostics GmbH, Mannheim, Germany). Sanger sequencing was carried out to identify mutations in the exons, flanking intronic regions and in the promoter of F13A1 using an ABI3130 Genetic Analyzer and Sequencing Analysis 5.4 software (Thermo Fisher Scientific, Carlsbad, CA) in a single patient with considerably low FXIII levels (<50%) at time point A and bleeding complications. All primers are available from the authors upon request.

### Clinical data

Neurological deficit of patients was determined by the National Institute of Health Stroke Scale (NIHSS) score^27^ on admission (before thrombolysis), 2 h, 24 h, and 7 days after thrombolytic therapy. Upon admission, all patients underwent computer tomography brain scan (CT) and computer tomography angiography (CTA) to ensure the diagnosis of acute ischemic stroke. Stroke etiology was classified according to the Trial of Org 10172 in Acute Stroke Treatment TOAST criteria^28^. A follow-up CT was performed for every patient 24 h after rtPA infusion. Hemorrhagic events on the follow-up CT scan were classified as symptomatic (SICH) or asymptomatic intracranial hemorrhage (aSICH) according to the European Cooperative Acute Stroke Study (ECASS) II criteria^13,29,30^. CT images taken before and 24 h after thrombolysis were analyzed simultaneously by 4 different investigators and the ASPECT scores were calculated^31,32^. For each patient the time of symptom onset, demographic and clinical characteristics (i.e.: cardiovascular risk factors, previous medication, neurological status, smoking habits) were recorded upon admission. Patients were followed and at 3 months after the event the modified Rankin score (mRS)^33^ was determined.

The following outcomes were investigated: 1/short-term functional outcome (by day 7 post-event), 2/long-term functional outcome (by the end of the 3^rd^ month post-event), 3/stroke-associated mortality by day 7, day 14 and by the end of the 3^rd^ month post-event, 4/the presence of therapy-associated symptomatic or asymptomatic intracranial bleeding (according to ECASS II criteria). Unfavorable short-term outcome was defined as an increase in NIHSS score by at least 4 points by day 7. Unfavorable long-term functional outcome was defined as an mRS score greater than 1 at 3 months post-event.

### Statistical analysis

Statistical analysis was performed using the Statistical Package for Social Sciences (SPSS, Release 22.0, Chicago, IL). Distribution of continuous variables was tested by the Kolmogorov-Smirnov test. Parametric variables were expressed as mean ± SD, non-parametric variables were presented as median (interquartile range) unless otherwise stated. In case of two-group analyses Student’s t-test or in case of non-parametric data Mann-Whitney U test was applied. Strength of association between variables was tested using Pearson’s correlation test. Multiple linear regression analysis was performed to adjust for parameters independently associated with FXIII levels. ANOVA using the Bonferroni correction was applied for multiple comparisons. Differences between categorical variables were assessed by the χ^2^ test. Logistic regression model was used to analyze the effect of FXIII levels and FXIII polymorphisms on various outcomes, results were expressed as odds ratio (OR) and 95% confidence interval (95% CI). When testing for independent risk factors of mortality, all potentially relevant risk factors (e.g. age, gender, stroke severity, smoking, CRP levels, NIHSS score on admission) were included in a multivariable backward conditional stepwise logistic regression model. Results were considered statistically significant when p < 0.05.

## Electronic supplementary material

Supplementary Table S1.
