# Supplementary material for: Low factor XIII levels after intravenous thrombolysis predict short-term mortality in ischemic stroke patients
Source: Sci Rep. 2018 May 16;8:7662. doi: 10.1038/s41598-018-26025-z (PMC5955963; doi:10.1038/s41598-018-26025-z)
Supplement: Supplementary file 1 — Supplementary Table S1. [file 41598_2018_26025_MOESM1_ESM.docx]

**Supplementary information**

**Low factor XIII levels after intravenous thrombolysis predict short-term mortality in ischemic stroke patients**

Edina Gabriella Székely^1^; Katalin Réka Czuriga-Kovács^2^; Zsuzsanna Bereczky^1^; Éva Katona^1^; Zoltán András Mezei^1^; Attila Nagy^3^; Noémi Klára Tóth^1^; Ervin Berényi^4^; László Muszbek^1^; László Csiba^2,5^; Zsuzsa Bagoly^1,5*^

*^1^Division of Clinical Laboratory Sciences, Department of Laboratory Medicine, Faculty of Medicine, University of Debrecen, Debrecen, Hungary; ^2^Department of Neurology, Faculty of Medicine, University of Debrecen, Debrecen, Hungary; ^3^Department of Preventive Medicine, Faculty of Public Health, University of Debrecen, Debrecen, Hungary; ^4^Department of Radiology, Faculty of Medicine, University of Debrecen, Debrecen, Hungary; ^5^MTA-DE Cerebrovascular and Neurodegenerative Research Group, Debrecen, Hungary*

^*^Correspondence to: Zsuzsa Bagoly, Division of Clinical Laboratory Sciences, Department of Laboratory Medicine, Faculty of Medicine, University of Debrecen, Debrecen, Hungary.

Address: Nagyerdei krt. 98, 4032 Debrecen, Hungary, Tel: +36 52431956; Fax: +36 52340011; e-mail: [bagoly@med.unideb.hu](mailto:bagoly@med.unideb.hu)

**Supplementary Table S1: Association between factor XIII (FXIII) levels and hemorrhage.**

|  | No hemorrhage (n=119) | aSICH (n=7) | SICH (n=6) | *P*-value*  no hemorrhage vs. SICH | *P*-value*  no hemorrhage vs. aSICH+SICH |
| --- | --- | --- | --- | --- | --- |
| Before thrombolysis (A)  FXIII activity (%)  FXIII-A_2_B_2_ (mg/l)  Immediately after thrombolysis (B)  FXIII activity (%)  FXIII-A_2_B_2_ (mg/l)  24 hours after thrombolysis (C)  FXIII activity (%)  FXIII-A_2_B_2_ (mg/l) | 126.2±36.1  22.4±7.8  125.3±33.4  21.4±6.7  116.3±34.6  19.6±6.4 | 131.5±22.5  23.5±4.9  123.6±25.1  22.4±6.3  129.9±30.1  21.7±3.8 | 117.8±50.3  19.1±7.1  114.3±52.2  19.2±10.1  102.5±54.6  16.4±10.1 | 0.588  0.315  0.444  0.437  0.460  0.350 | 0.930  0.674  0.547  0.807  0.712  0.903 |

Results are expressed as mean ± standard deviation. n, number of patients; aSICH, asymptomatic intracranial hemorrhage; SICH, symptomatic intracranial hemorrhage; * ANOVA with Bonferroni post hoc test
